# Architectural Insight into Inovirus-Associated Vectors (IAVs) and Development of IAV-Based Vaccines Inducing Humoral and Cellular Responses: Implications in HIV-1 Vaccines

**DOI:** 10.3390/v6125047

**Published:** 2014-12-17

**Authors:** Kyriakos A. Hassapis, Dora C. Stylianou, Leondios G. Kostrikis

**Affiliations:** Department of Biological Sciences, University of Cyprus, 75 Kallipoleos Avenue, 1678 Nicosia, Cyprus; E-Mails: hasapis.kyriakos@ucy.ac.cy (K.A.H.); stylianou.c.dora@ucy.ac.cy (D.C.S.)

**Keywords:** HIV-1 vaccine, inovirus, inovirus display, phage display, viral vectors, antigen display, inovirus-associated vectors

## Abstract

Inovirus-associated vectors (IAVs) are engineered, non-lytic, filamentous bacteriophages that are assembled primarily from thousands of copies of the major coat protein gp8 and just five copies of each of the four minor coat proteins gp3, gp6, gp7 and gp9. Inovirus display studies have shown that the architecture of inoviruses makes all coat proteins of the inoviral particle accessible to the outside. This particular feature of IAVs allows foreign antigenic peptides to be displayed on the outer surface of the virion fused to its coat proteins and for more than two decades has been exploited in many applications including antibody or peptide display libraries, drug design, and vaccine development against infectious and non-infectious diseases. As vaccine carriers, IAVs have been shown to elicit both a cellular and humoral response against various pathogens through the display of antibody epitopes on their coat proteins. Despite their high immunogenicity, the goal of developing an effective vaccine against HIV-1 has not yet materialized. One possible limitation of previous efforts was the use of broadly neutralizing antibodies, which exhibited autoreactivity properties. In the past five years, however, new, more potent broadly neutralizing antibodies that do not exhibit autoreactivity properties have been isolated from HIV-1 infected individuals, suggesting that vaccination strategies aimed at producing such broadly neutralizing antibodies may confer protection against infection. The utilization of these new, broadly neutralizing antibodies in combination with the architectural traits of IAVs have driven the current developments in the design of an inovirus-based vaccine against HIV-1. This article reviews the applications of IAVs in vaccine development, with particular emphasis on the design of inoviral-based vaccines against HIV-1.

## 1. Structural and Biological Insights Into Filamentous Viruses

**Figure 1:**
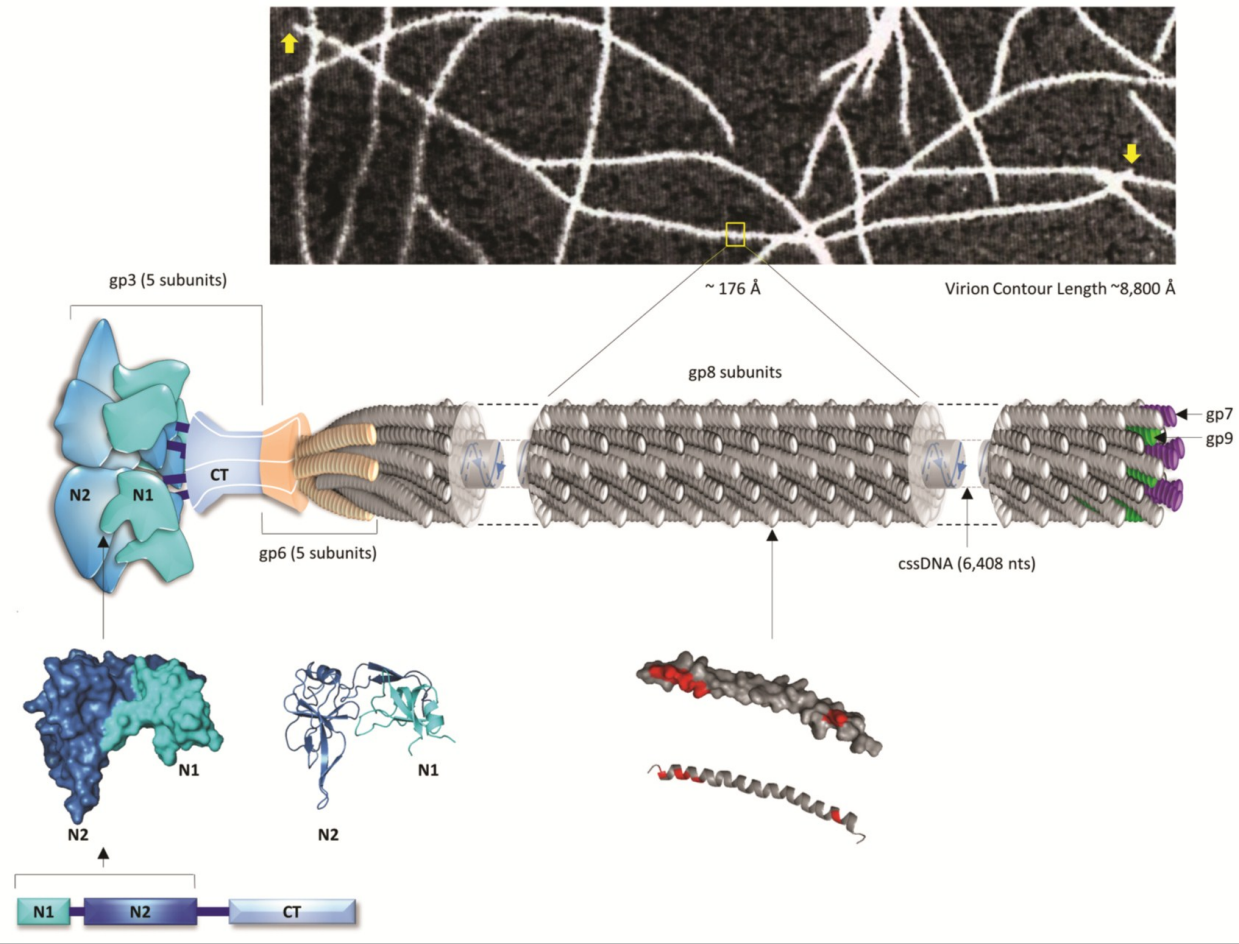
A 3D scale schematic model of an end-to-end Ff (fd) inoviral virion. The model is based on published physical data including the determined helical parameters of the major coat protein gp8 and the X-ray structure of the N1-N2 domains of the minor coat protein gp3. The model shows the relative location of the circular single-stranded DNA (cssDNA) genome (6408 nucleotides long, illustrated as blue ribbons), some structural details of the outer virion capsid (major coat protein pg8) and the four minor coat proteins (gp6, gp3, gp7 and gp9) present at the ends of the virion. On top, a digital scanning transmission electron micrograph (STEM) of unstained fd virus, prepared by the wet-film technique according to previously established procedures of the Brookhaven STEM facility [32,33,34]. The ends of one complete virion are designated by arrows. The data were collected in collaboration with L.A. Day and J. S. Wall at the Brookhaven National Laboratory, Upton New York. Under these STEM conditions fd virions are about 8800 Å long and about 65 Å in diameter [3]. In the middle, a proposed end-to-end scale 3D diagram of fd virion is presented. The entire fd virion is composed of about 2700 subunits of gp8 with the exception of its two ends. Architectural details of an axial slab 176 Å long (about 1/50 of the virion length) consisting entirely of subunits of major coat protein gp8. The structure of the 50-amino-acid-long and extended α-helical gp8, shown below in both surface and ribbon images, is presented in the virion model as a cylindrical stack of 25 gray disks about 70 Å long and 10 Å in diameter. The images of gp8 were derived from coordinates of RCSB PDB database accession number 2cOW [35] using PyMOL [36]. The gp8 subunits are arranged with a helical symmetry that includes a two-fold screw axis and a five-fold rotation axis, consisting of two pentamers of pg8 [28,29,30,35]. The two pentamers are architecturally related to each other by a translation of about 16 Å along the virion axis and a rotation of 36° about the axis [28]. The proximal end of the virion, shown on the left, is composed of five copies of each of the minor coat proteins gp6 and gp3 (for a recent review see [4]). The proximal end is modeled based on partial information known about the structures of gp6 and gp3. Specifically, the N-terminal portion of gp6 was modeled following the helical parameters of gp8, based on protein sequence homology between the two [23,24,25]. Five copies of gp3 subunits were modeled based on structural information of the N1-N2 domains. The images of the N1-N2 domains of gp3 are shown below and were derived from coordinates of RCSB PDB database accession number 1g3p [36] using PyMOL. The domain organization of gp3 is also shown. The distal end of the virion (right) consists of five copies of each minor coat proteins gp7 and gp9, modeled following the helical parameters of gp8 according to a previously published model [25].

Filamentous bacterial viruses are a group of thread-like viruses containing single-stranded DNA genomes. Collectively, they constitute the genus *Inovirus* in the family *Inoviridae*, the terms deriving from the Greek word *Ίνα* for filament [1,2,3], and they are commonly called filamentous bacteriophages. There are over 50 different known individual species of filamentous viruses; the majority of them capable of infecting Gram-negative bacteria. The complex interaction between filamentous phages and their bacterial hosts is specified by receptor organelles that are usually encoded by transmissible plasmids [1,4]. One of the most intriguing features of inoviruses is that they are assembled at the host membrane, where the major capsid protein subunits replace the single-stranded DNA binding protein, and progeny virions are continuously extruded into the medium without killing the infected cell, giving rise to titers of up to 10^13^ virions per milliliter of liquid culture [5,6]. The high virus production is associated only with a mild retardation of the host’s growth, which gives rise to the formation of opaque plaques on bacterial lawns. In this sense, filamentous viruses bear a resemblance to symbiotic non-pathogenic animal viruses rather than *phages*, the term coming from the Greek word *φάγος* for destroyer. Inovirus virions are flexible and slender cylindrical filaments [2,3] less than 10 nm in diameter and in the order of 1000 nm in length (see details in Figure 1). Each virion has several thousand identical major capsid or coat protein subunits packaging a circular single-stranded DNA molecule. Each virion also has a few specific minor proteins at each end, those at one end (proximal end) for attachment during infection, and those at the other end (distal end) for nucleation and initiation of the assembly process at the host’s membrane.

The number of species of *inovirus* isolated and characterized in different parts of the world is rather large [1]. Among *E. coli* inoviruses, the best-studied and most-exploited is Ff, a group of closely related viruses (fd, f1 and M13) (for review see [4,6]) that infect male (F^+^) strains of *E. coli*. All Ff have almost identical DNA and protein sequences, gene organization, and most other structural parameters [7,8,9]. Fd, M13 and f1 differ in their genomes at only about 100 positions out of 6408 nucleotides for f1 and fd or 6407 nucleotides for M13. The genetics and life cycle of three viruses f1, fd and M13 have been studied extensively and we know a great deal about them. The Ff genome contains ten tightly arranged genes and a non-coding intergenic region, which contains the packaging signal [10,11,12], the (+) and (−) origins of DNA replication, and the major rho-dependent transcriptional terminator (for recent review see [4]). Five of the ten viral genes encode proteins found in the virion (g3, g6, g8, g7 and g9). Genes 3 and 6 are found on the proximal end of the virion and are essential for infectivity and stabilization, whereas, gene 7 and gene 9 proteins, gp7 and gp9, respectively, are located at the distal end of the virion and are responsible for the initiation assembly [13,14,15]. In the end-to-end model illustrated in Figure 1, both the proximal (gp7 and gp9) and distal (gp3 and gp6) minor coat protein subunits maintain the fivefold axial symmetry of the major coat protein gp8 subunits along the virion.

The life cycle of Ff filamentous viruses starts with the adsorption of the virus to the tip of the F^+^ specific pilus of *E. coli*. Attachment takes place by means of an adsorption structure composed of five copies of gp3, located at the proximal end of the virion (see Figure 1), mainly through sequential binding of the gp3 N2 domain with the tip of the F pilus and the N1 domain with the periplasmic domain III of TolA (for recent review see reference 4). After adsorption, the virus is drawn to the surface of the cell where the major coat protein gp8 subunits become associated with the inner membrane of the cell [16,17,18] and the circular single-stranded DNA (cssDNA) is released into the cytoplasm. Inside the cytoplasm, the cssDNA is converted into a parental double-stranded replicative form (RF). Ff inoviruses replicate their genome by a rolling-circle mechanism. Consecutive transcription from the RF DNA, and translation as well an asymmetric single strand DNA synthesis lead to an intracellular pool of viral precursor complexes, which contain viral single-stranded DNA molecules bound with gp5 protein subunits, except a small hairpin loop that serves as the packaging signal for virion assembly [10,11,12]. The major coat protein gp8 subunits are synthesized with a signal peptide sequence that facilitates their transport to and insertion into the bacterial membrane, where they are cleaved by signal peptidase. After cleavage, the mature coat protein is left spanning the membrane with its C terminus in the cytoplasm and the N terminus outside the cytoplasm in the periplasm [19,20,21]. The assembly of filamentous viruses takes place at the membrane where the gp5 subunits are replaced by gp8 subunits. The first step of the assembly sequence is the binding of the packaging signal, a site of about 30 nucleotides that forms a hairpin loop, with presumably five subunits of each of the minor coat proteins gp7 and gp9 [22,23,24,25,26]. Virus assembly proceeds as single-stranded DNA passes through the membrane and more mature coat protein gp8 subunits are added until the entire DNA molecule is packaged. At the distal end of the virion, five copies of each of gp6 and gp3 are added and the complete virion is released into the medium [23,27]. The assembly of inoviruses on the bacterial inner membrane is a harmonized sequential process that involves a variety of interactions between viral-encoded proteins (gp1, gp4 and gp11) and host-encoded proteins, without killing the host bacterium (reviewed by [4]).

The structure of the Ff virus has been extensively studied by a number of laboratories in the last five decades. However, despite all of the efforts, the structure has not been completely determined and some critical questions remain unanswered. The major difficulty is that these viruses cannot be crystallized. They can be oriented in fibers suitable for X-ray fiber diffraction studies, but these are not crystals. Interpretations of the fiber diffraction patterns together with a number of physicochemical measurements, have shown that the major coat protein gp8 subunits have a five-start helical symmetry (5-fold rotation axis) and are referred to as Class I [28,29,30] strictly based on the fundamental symmetry of the protein subunits helices as determined by fiber diffraction (reviewed by [2]). On the other hand, the structure of the packaged ssDNA molecule in the virion, including its helical symmetry and the interactions with the protein sheath, is one of the least understood aspects. The structure of the DNA inside these viruses cannot be determined by conventional X-ray fiber diffraction techniques, partly because of the low DNA content in the virions. Theoretical studies have demonstrated that the single-stranded DNA molecule is uniquely packaged inside the Ff virion by predominant electrostatic interactions [3,31].

## 2. Inovirus Associated Vectors

**Figure 2:**
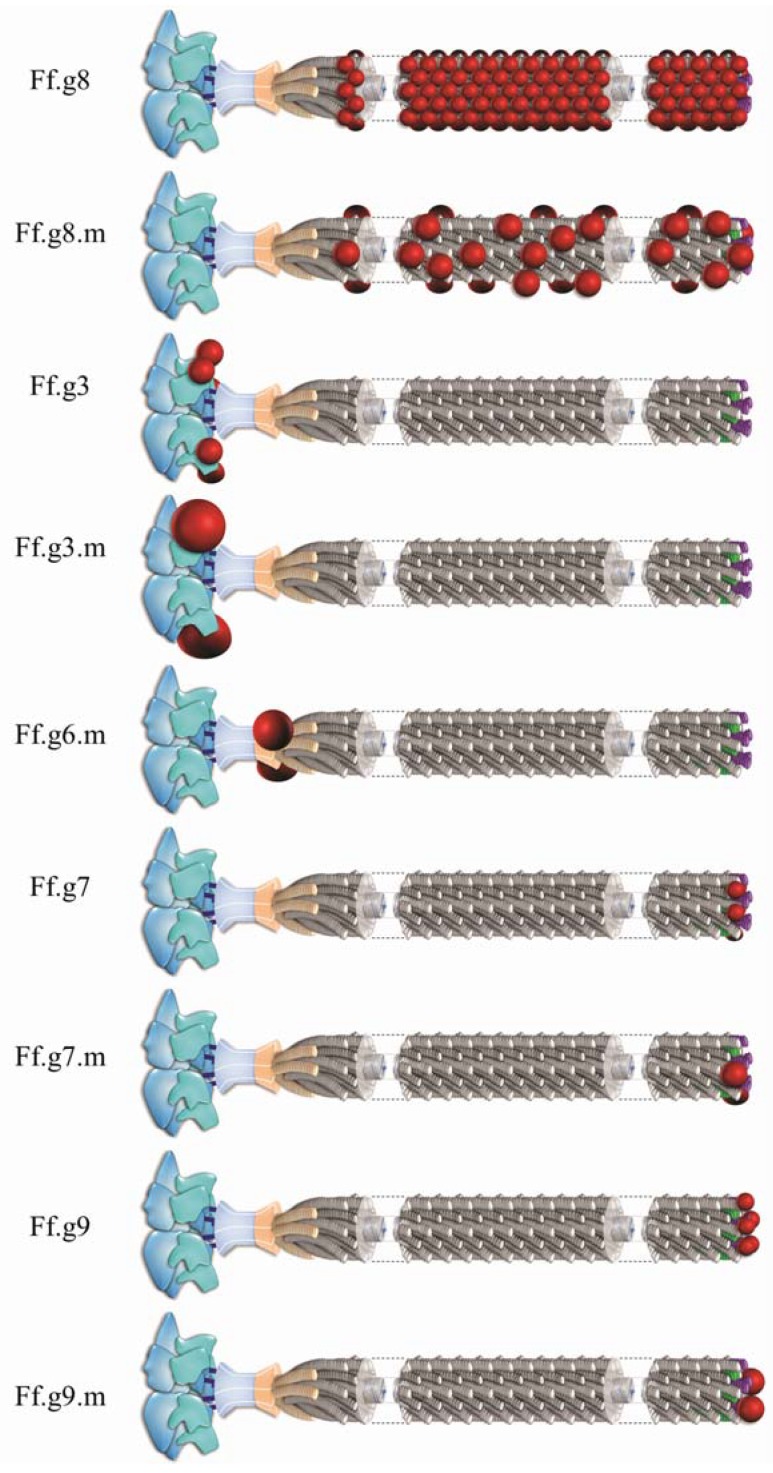
Schematic representations of antigen display on the surface of Ff inovirus-associated vectors (IAVs). Foreign antigens are shown as red spheres. The designation on the left denotes the inoviral gene, which can be genetically modified to express an antigen on the outer architecture of the virion. IAVs that contain both the wild type and antigen display capsid proteins are designated by “m” which indicates that the virion is a mosaic. Each Ff virion contains about 2700 copies of major capsid protein gp8, and five copies of each of the minor capsid proteins, gp3, gp6, gp7 and gp9.

**Figure 3:**
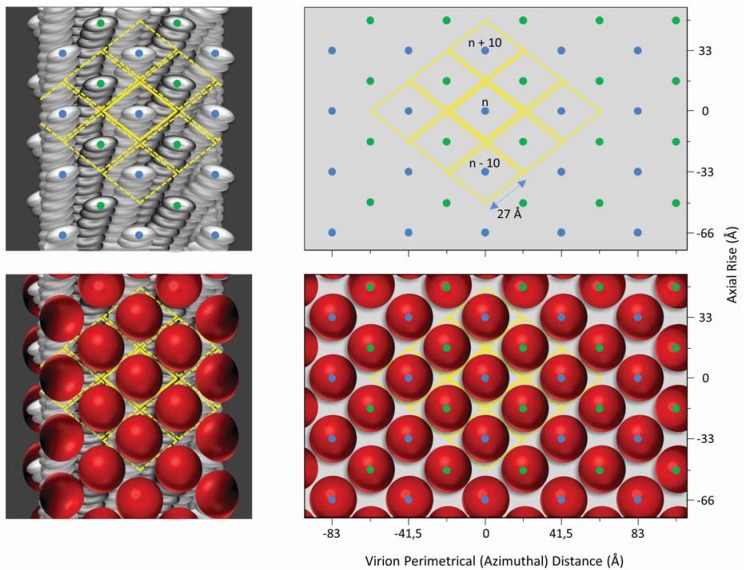
Surface lattice diagrams of wild-type Ff the Ff.g8 inovirus-associated vector. Left, virion surface models of axial slabs about 130 Å long of wild-type Ff (top) and Ff.g8 inovirus-associated vector (bottom) showing the major coat protein gp8 subunits arranged with a combined five-fold rotation axis and an approximate two-fold screw axis [28]. Right, the corresponding surface lattices, identical to those previously published [30]. The lattice diagrams show the relative position of each gp8 subunit on the outer virion surface. The five gp8 subunits of each of the two interlocking pentamers constituting the helical symmetry of the virion are indicated by blue and green dots respectively. The relative virion surface area (about 1400 Å2) associated with each gp8 subunit is marked in yellow. The virion perimetrical (azimuthial) distance is calculated based on a virion diameter of about 65 Å. The displayed antigens, represented by red spheres, are arranged on the surface of the Ff.g8 inovirus-associated vector according to helical symmetry of the virion outer architecture (bottom).

The easy genetic manipulation of inoviruses and the possibility of inserting random oligonucleotides into their genome set the foundation for inovirus display (phage display) technology [37,38]. Expression of these genetically modified inoviruses results in the presentation of oligopeptides as fusion proteins on the surface of the virion and are herein termed IAVs for inovirus-associated vectors. IAVs can be modified to express an oligopeptide on either all or on some copies of a particular capsid protein. One possibility is to insert an oligonucleotide sequence of interest in the viral genome to create a fusion with capsid protein gp3, gp7, gp8 or gp9, so that the oligopeptide is displayed on every copy of the capsid protein. Alternatively, a phagemid vector can be used, which carries an extra copy of a capsid protein to which the oligonucleotide is fused. Coinfection of bacterial hosts with the phagemid vector and a replication deficient helper phage, that carries the wild type capsid protein, would result in mosaic inovirus particles. That is, they will contain copies of both the wild type coat protein and the recombinant protein that contains the oligopeptide of interest [39]. Non-mosaic and mosaic IAVs that display a peptide on gp3 or gp8 have been recently reviewed [4] while IAVs that display a peptide on gp7 or gp9 have been reviewed elsewhere [40,41,42]. In contrast to the other four-capsid proteins, gp6 capsid protein has only been utilized for the production of mosaic virions [43,44,45]. Figure 2 illustrates the display of an antigen on each of the five capsid proteins of an IAV as indicated in published literature. It also introduces a new terminology to denote the gene to which the oligopeptides are fused to and whether the virion is a mosaic. To display many copies of an oligopeptide on an IAV, the ideal capsid protein to utilize is gp8. The resulting non-mosaic IAV can display a peptide on each of the approximately 2700 copies of gp8 on its capsid surface. The tradeoff, however, is a significant limitation in the size of the peptide: only peptides up to 6 amino acids may be displayed without distorting the assembly of the virus (see Figure 2 and Figure 3). This size restriction of the displayed peptide may be overcome by generating a mosaic IAV that displays the foreign peptide in only a minority of gp8 on the viral surface [38]. With regards to the display of peptides on gp3, it is possible through mosaic IAVs to present even a whole protein on the viral surface [46]. Although in such a case the protein is expected to be present in up to five copies per virion, studies show that virions carry none, or just one copy of the protein on their surface [39].

Random Peptide Libraries (RPL) is one common application of IAVs, where random oligopeptides are displayed on different clones of an inovirus particle. The vast diversity of an RPL depends on the size of the oligopeptide where the complexity of an RPL increases exponentially as the size of the oligopeptide increases. RPLs can subsequently be used in many applications including the identification of peptide ligands by receptors, the mapping of substrate sites for enzymes, and the creation of antibody peptide libraries. These applications are reviewed elsewhere [38,46,47]. Inovirus display technology has also been used for epitope mapping and vaccine design purposes. RPLs can be used to characterize the epitope of an antibody of interest through biopanning with a monoclonal antibody of interest, which can result in the isolation of mimotopes; these are oligopeptides that mimic the native antibody epitope. The selected recombinant inoviruses that carry mimotopes can then be isolated in order to determine the DNA and amino acid sequence of the displayed oligopeptides. DNA sequencing of such inserts as well as structure prediction analysis can potentially identify the previously unknown target of an antibody. Besides antibody characterization, inoculation of recombinant inoviruses isolated through this approach can potentially be used as vaccine carriers. For example, if a neutralizing antibody against a pathogen is used to screen an RPL, the selected peptides (fused to inoviruses) would mimic the original antibody epitope. Vaccination of animals with these inoviruses could ultimately induce the production of similar antibodies by the vaccinated individual, offering protection from infection against the pathogen. Inovirus display technology has been successfully applied in the development of vaccines against various pathogens (Table 1 and Table 2). The potential for inovirus display technology to facilitate the mapping of antibody epitopes is of great importance, especially in the case of HIV-1. Epitopes of broadly neutralizing monoclonal anti-HIV-1 antibodies could be rare, vulnerable spots on the surface of a frequently mutating virus such as HIV-1 and therefore, their identification and further study could lead to new drug therapies or vaccine targets. This review focuses particularly on the applications of inovirus display technology that utilizes capsid proteins gp3 and gp8, as those have been used in vaccine development.

**Table 1:** Summary of Inovirus-Based Vaccines Against Pathogens Causing Infectious Diseases.

Pathogens ^a^	Inoviral Vector ^b^	Antigen ^c^	Study Animal Model	Type of Immune Response ^d^	Protection of Vaccinated Animals against Challenge with the Pathogen ^e^	References	
Viral							
HBV	M13.g8.m	Hepatitis B Surface Antigen	Mice	Specific humoral	ND	Folgori *et al.*, 1994	[48]
HBV	M13.g8.m	Hepatitis B Surface Antigen	Mice	Specific cellular (CD8)	ND	Wan *et al.*, 2001	[49]
HIV-1	fd.g3	gp120 (V3 loop)	Rabbits	Specific humoral	ND	Keller *et al.*, 1993	[50]
HIV-1	M13.g8.m	Unknown	Mice	Specific humoral	ND	Scala *et al.*, 1999	[51]
HIV-1	fd.g8.m	gp120 (CD4 binding site)	Mice, Rabbits	Non-specific humoral	ND	Zwick *et al.*, 2001	[52]
HIV-1	M13.g8.m	Unknown	Rhesus macaques	Specific humoral	Partial	Chen *et al.*, 2001	[53]
HIV-1	fd.g3	gp120 (CD4 binding site)	Mice	Specific humoral	ND	Dorgham *et al.*, 2005	[54]
HIV-1	M13.g3	gp120 (CD4 binding site)	Rabbits	Specific humoral	ND	Wilkinson *et al.*, 2007	[55]
HIV-1	M13.g3	Unknown	Mice	Specific humoral	ND	Rodriguez *et al.*, 2007	[56]
HIV-1	M13.g3	Unknown	Mice	Specific humoral	ND	Humbert *et al.*, 2007	[57]
HIV-1	M13.g3	Unknown	Mice	Specific humoral	ND	Humbert *et al.*, 2008	[58]
HIV-1	fd.g8.m	Carbohydrates	Rabbits	Non-specific humoral	ND	Menendez *et al.*, 2008	[59]
HIV-1	M13.g8.m	gp120 (V3 loop)	Mice	Specific cellular (CD8)	ND	Pedrosa-Roldan *et al.*, 2009	[60]
HIV-1	M13.g8.m	gp120 (V3 loop)	Mice	Specific humoral	ND	Charles-Nino *et al.*, 2011	[61]
HIV-1	M13.g3	gp41 (MPER)	Mice, Rabbits	Specific humoral	ND	Rodriguez *et al.*, 2011	[62]
HIV-1	M13.g3	gp120 (V3 loop)	Rabbits	Specific humoral	ND	Gazarian *et al.*, 2013	[63]
HPV	fd.g8.m	E7	Mice	Specific humoral	ND	Lidqvist *et al.*, 2008	[64]
HSV-2	fd.g8.m	Glycoprotein G2	Mice	Specific humoral	Significant	Grabowska *et al.*, 2000	[65]
Influenza A	M13.g3	Hemagglutinin Antigen	Mice	Specific humoral	Significant	Zhong *et al.*, 2011	[66]
Neurotropic Murine Coronavirus	fd.g8.m	Surface Glycoprotein	Mice	Specific humoral	Partial	Yu *et al.*, 2000	[67]
Rabies virus	M13.g3	Rabies Viral Glycoprotein	Mice	Specific humoral	ND	Houimel *et al.*, 2009	[68]
RSV	fd.g3	Glycoprotein G	Mice	Specific humoral	Complete	Bastien *et al.*, 1997	[69]
Protozoan							
*Plasmodium falciparum*	fd.g3	CSP	Mice, Rabbits	Specific humoral	ND	Stoute *et al.*, 1995	[70]
*Plasmodium falciparum*	F1.g3	CSP	Mice, Rabbits	Specific humoral	ND	de la Cruz *et al.*, 1988	[71]
*Plasmodium falciparum*	fd.g8.m	CSP	Rabbits	Specific humoral	ND	Greenwood *et al.*, 1991	[72]
Fungal							
*Candida albicans*	fd.g8.m	Heat Shock Protein 90	Mice	Specific humoral and cellular (CD4)	Partial	Yang *et al.*, 2005	[73]
*Candida albicans*	fd.g8.m	Heat Shock Protein 90	Mice	Specific humoral and cellular (CD4)	Partial	Wang *et al.*, 2006	[74]
Worm							
*Fasciola hepatica*	M13.g3	Cathepsin L	Sheep	Specific humoral	Partial	Villa-Mancera *et al.*, 2008	[75]
*Schistosoma japonicum*	M13.g3	Ferritin of Sj	Mice	Specific humoral	Partial	Tang *et al.*, 2004	[76]
*Schistosoma japonicum*	M13.g3	Epitope of mAb SSJ14	Mice	Specific humoral and cellular (CD4)	Significant	Wang *et al.*, 2006	[77]
*Schistosoma japonicum*	M13.g3	Sj338	Mice	Specific humoral	Partial	Wu *et al.*, 2006	[78]
*Taenia solium*	M13.g8.m, M13.g3	GK1, KETc1, KETc7, KETc12	Guinea Pigs	Non-specific humoral and specific cellular (CD4)	Significant	Manoutcharian *et al.*, 2004	[79]
*Trichinella spiralis*	M13.g3	Ts87	Mice	Specific humoral	Partial	Gu *et al.*, 2008	[80]
*Trichirella spiralis*	M13.g3	Ts Paramyosin	Mice	Specific humoral	Partial	Wei *et al.*, 2011	[81]

^a^ HBV, Hepatitis B Virus; HIV-1, Human Immunodeficiency Virus Type 1; HPV, Human Papilloma Virus; HSV-2, Herpes Simplex Virus Type 2; RSV, Respiratory Syncytial Virus. ^b^ F1, M13, fd denote the name of Filamentous Viral Vector; g8 and g3 denote the viral capsid genes in which antigens are displayed as fusion proteins; m, mosaic. It denotes that the inoviral vaccine contains both wild type and antigen-displayed capsid proteins. ^c^ gp120, HIV-1 glycoprotein 120; Unknown, a pool of inoviruses displaying random peptides biopanned with polyclonal antibodies from sera of HIV-1-infected individuals; gp41, HIV-1 glycoprotein 41; MPER, membrane-proximal external region of gp41; CSP, Circumsporozoite Surface Protein. For HIV-1 antigens gp120 and gp41, the epitopes are provided in parentheses. ^d^ CD8 and CD4 T cell responses are indicated in parentheses where the information is available in the corresponding references. ^e^ ND, Not Determined.

**Table 2:** Summary of Inovirus-Based Vaccines Against non-Infectious Diseases.

Diseases	Inoviral Vector ^a^	Antigen ^b^	Study Animal Model	Type of Immune Response ^c^	Protection of Vaccinated Animals against disease progression ^d^	References	
Cancer							
Colorectal cancer	M13.g3	EGFR	Mice	Specific humoral	ND	Riemer *et al.*, 2005 A	[82]
Melanoma	fd.g8.m	HMW-MAA	Mice	Specific humoral	ND	Riemer *et al.*, 2005 B	[83]
Melanoma	fd.g3	HMW-MAA	Mice	Specific humoral	ND	Luo *et al.*, 2005	[84]
Melanoma	fd.g8.m	MAGE A1	Mice	Specific cellular (CD8 and CD4)	Significant	Fang *et al.*, 2005	[85]
Melanoma	Ff.g8 or Ff.g8.m	HMW-MAA	Rabbits	Specific humoral	ND	Wagner *et al.*, 2005	[86]
Melanoma	fd.g3	HMW-MAA	Rabbits	Specific humoral	ND	Luo *et al.*, 2006	[87]
Melanoma	F1.g8.m, M13.g3	HMW-MAA	Mice, Rabbits	Specific humoral only in mice	ND	Latzka *et al.*, 2011	[88]
Murine mastocytoma P815	M13.g8.m	P1A	Mice	Specific cellular (CD8 and CD4)	Significant	Wu *et al.*, 2002	[89]
Various types of cancer	M13.g3	Epitope of mAb BAT	Mice	Specific humoral and cellular (CD8)	Significant	Hardy *et al.*, 2005	[90]
Various types of cancer	M13.g3	EGFR	Rabbits	Specific humoral	ND	Hartman *et al.*, 2010	[91]
Various types of cancer	M13.g3	VEGF	Mice	Specific humoral	ND	Li *et al.*, 2013	[92]
Alzheimer’s							
	fd.g8.m, fd.g3	EFRH	Guinea pigs	Specific humoral	ND	Frenkel *et al.*, 2000	[93]
	fd.g8.m	EFRH	Mice	Specific humoral	Significant	Frenkel *et al.*, 2003	[94]
	fd.g8.m, fd.g3	EFRH	Mice	Specific humoral	Partial	Lavie *et al.*, 2004	[95]
	fd.g8.m, fd.g3	EFRH	Mice	Specific humoral	Partial	Solomon *et al.*, 2007	[96]

^a^ F1, M13, fd denote the name of Filamentous Viral Vector; g8 and g3 denote the viral capsid genes in which antigens are displayed as fusion proteins; m, mosaic. It denotes that the inoviral vaccine contains both wild type and antigen-displayed capsid proteins. ^b^ EGFR, Epidermal Growth Factor Receptor; HMW-MAA, High Molecular Weight Melanoma-Associated Antigen; MAGE A1, Melanoma Antigen A1; VEGF, Vascular Endothelial Growth Factor; EFRH, continuous peptide consisting of amino acids E, F, R and H, which is located at positions 3–6 within β-amyloid peptide and represents the sequential epitope of mAbs 6C6 and 10D5 [97]. ^c^ CD8 and CD4 T cell responses are indicated in parentheses where the information is available in the corresponding references. ^d^ ND, Not Determined.

## 3. Inovirus Display Technology in Vaccine Design against Non-HIV-1 Diseases

IAVs are effective vaccine carriers and, as shown in Table 1 and Table 2, they have been used successfully in numerous vaccine development studies. They have been utilized in the development of vaccines against a wide variety of viral, protozoan and worm parasites and also against non-infectious diseases like Alzheimer and various types of cancer. All these attempts can be divided in two main sub-categories. The first sub-category, inovirus display technology was used to screen RPLs with monoclonal antibodies and then to select the immunogenic peptides of interest. The selected peptide was used as a vaccine in its soluble form or in conjugation with carrier proteins [55,66,70,81,82,83,84,86,87,88,90,91,92,98]. In the second sub-category, similar to the first sub-category, inovirus display technology has been used for epitope mapping and isolation, but in this case, inoviruses were also used as the vaccine carriers for the immunogenic peptides [48,49,50,51,52,53,54,56,57,59,60,61,62,63,64,65,67,68,69,71,72,73,74,75,76,77,78,79,80]. In contrast to IAVs, soluble peptides have the disadvantage of being less stable than the same peptides fused to inoviral particles. Soluble peptides have a flexible 3D structure and thus, do not always retain the 3D structure of the desirable epitope. As a result, soluble peptides, unlike inovirus-bound peptides, are less capable of inducing the production of the desirable antibodies [99]. Additionally, the inoviral vectors displaying peptides are highly immunogenic. Their high immunogenicity is reinforced by the ability of inoviruses to display multiple copies of peptides on their surface. Additionally, inoviruses are known for their structural simplicity, which allows the immune system to focus selectively on the displayed peptides and not on the viral carrier [100]. Furthermore, since inoviruses can replicate in *E. coli* cultures, the cost of vaccine production is low. In summary, IAVs can be used as efficient and cost effective vaccine carriers.

A large number of research studies have focused on the application of inovirus-based vaccines against infectious diseases. A common approach in many of these efforts was to vaccinate animals with inoviruses and to then challenge them with specific pathogens, in order to assess the level of protection against the pathogens. Three of these studies focused on immunization against viral parasites. In 1997, Bastien *et al.* fused a 15-mer linear epitope of Human Respiratory Syncytial Virus (RSV) glycoprotein G on inovirus gp3 and used the recombinant inovirus to vaccinate mice [69]. This resulted in a specific humoral response, with the vaccinated animals having complete protection from challenge with the RSV virus [69]. In 2000, Grabowska *et al.* used monoclonal antibodies against Herpes Simplex Virus type 2 (HSV-2) glycoprotein G2 to screen 15-mer RPLs [65]. The selected recombinant inoviruses were used to vaccinate mice, resulting in a specific humoral response. A high survival rate of vaccinated mice after challenge with a lethal dose of the virus was observed, and the level of protection was proportional to the dose of the inoviral vaccine [65]. Additionally, in 2000, Yu *et al.* used monoclonal antibodies against the surface glycoprotein of Neurotropic Murine Coronavirus to screen various RPLs [67]. A selected clone displaying a 13-mer peptide induced a humoral immune response but no cellular response in mice. Even so, after lethal virus challenge, three out of six mice survived. [67]. Besides viral infections, inoviral vaccine research has also been applied for systemic candidiasis, a fungal infection caused by *Candida albicans.* In two separate studies, a specific six amino acid peptide epitope of the fungal heat shock protein 90 was displayed as a fusion with the inoviral coat protein gp8 [73,74]. After infection with the parasite, mice immunized with the recombinant inoviruses had fewer colony forming units of *Candida albicans* in the kidney and a longer lifespan [73]. The use of inovirus as a vaccine carrier was particularly successful against *Taenia solium*, a parasitic worm that uses pigs as intermediate hosts and causes neurocysticercosis, a parasitic disease of the central nervous system, in humans [79]. In 2004, Manoutsarian *et al.* fused four antigenic peptides (GK1, KETc1, KETc7, KETc12) to inoviruses and the cocktail of recombinant inoviruses was used to vaccinate pigs; as a result, a specific cellular response was induced. Vaccination of pigs protected them against challenge with the pathogen: 1/3 of pigs were totally protected and 2/3 had reduced number of cysticerci [79]. Based on these results, large-scale vaccination of 1047 rural pigs in 16 villages of central Mexico was conducted in 2008. The immunization was successful since the vaccine conferred significant protection against the parasite. Furthermore, this inovirus-based vaccine was more economical compared with to another vaccine made of synthetic peptides. The study was particularly important, not only due to the large scale vaccination attempt with inoviruses, but also due to its effectiveness in significantly reducing the number of cysticerci in the vaccinated animals [101]. Efforts were also made to develop vaccines against the worm *Schistosoma japonicum.* In 2004, Tang *et al.* screened a 12-mer RPL with polyclonal serum from infected mice [76]. The selected recombinant inoviruses induced a specific humoral response, which conferred partial protection from parasite challenge in the vaccinated mice [76]. Following this study, in 2006, Wang *et al.* used the monoclonal antibody SSj14, which targets the parasite to screen a 12-mer RPL [77]. The recombinant inoviruses induced both humoral and cellular responses that significantly protected the vaccinated mice against the worm infection [77]. Additionally, in 2006, Wu *et al.* used polyclonal serum from infected rabbits to screen a 12-mer RPL [78]. A humoral response was induced in the vaccinated mice, which conferred partial protection from the parasite [78]. In 2008, Villa-Mancera *et al.*, produced a vaccine against *Fasciola hepatica*, *by* screening a previously constructed 12-mer inovirus RLP with an anti-cathepsin L monoclonal antibody [75]. Although immunization of sheep with the selected recombinant inoviruses induced a weak, specific humoral response after challenge with the parasite, the vaccinated animals had a remarkable reduction in worm burden compared to controls [75]. An inovirus-based vaccine has been constructed against the parasitic worm *Trichinella spiralis.* In this case, Gu *et al.* used a monoclonal antibody against rTs87 antigen to screen a 12-mer RPL [80]. As a result, a humoral response was induced and the vaccinated mice gained partial protection after challenge against the parasite [80]. In summary, the results of the above studies show that the construction of an efficient inovirus-based vaccine that confers protection to the vaccinated animals against the infectious pathogen is achievable through the induction of humoral or cellular immune response or both.

As previously mentioned, inovirus display technology has also been used for the design of vaccines against non-infectious diseases and in some cases, the capability of the vaccine to limit the progression of the disease was evaluated. In 2005, Fang *et al.* displayed an epitope of the Melanoma Antigen A1 (MAGE A1) in the surface of inovirus fused on gp8 [85]. This resulted in an induction of cellular immune response against the melanoma tumor and in the significant inhibition of tumor growth in the vaccinated mice. In addition, there was an important increase in the survival rate of vaccinated animals [85]. Similar results were obtained in 2002 by Wu *et al.* in an effort to design a vaccine against murine mastocytoma P815 [89]. An epitope of the P1A tumor antigen was fused to the inoviral surface and the vaccinated mice gained significant protection against tumor growth. Moreover, there was a significant increase in survival rate due to an anti-tumor cellular response that was induced [89]. Some important efforts have also been made for the design of an inovirus-based vaccine against Alzheimer’s disease. The main target of these vaccines was the induction of antibodies against β-amyloid plaques. In these studies, the antigenic epitope that was displayed in the inoviral surface was the epitope EGFR, which consists of the four amino acids E, G, F and R and it is part of the β-amyloid peptide. In mice immunized with recombinant inoviruses, the researchers observed a reduction in β-amyloid plaque burden and a specific humoral response [93,94,95,96]. Collectively, these studies show that it is possible to protect vaccinated animals against disease progression, thus alluding to the promising use of such vaccines against non-infectious diseases in humans.

In summary, the utilization of inoviral vectors for vaccine development against infectious and non-infectious non-HIV-1 diseases has produced significant and promising results. First, in the majority of cases, the vaccine was successful since it induced specific humoral or cellular response, or both. Furthermore, in many cases, there was an attempt to evaluate the efficacy of the vaccine after challenge against the pathogen in vaccinated animals. In all cases, the vaccine could provide partial, significant or even complete protection against the pathogen. This established the effectiveness of the use of inoviruses as vaccine vectors.

## 4. HIV-1 Inovirus-Based Vaccines

Our knowledge of HIV-1 neutralization epitopes initiated from the isolation of several neutralizing monoclonal antibodies (2F5, 4E10, b12 and 2G12) that were described between 1993 and 1994 [102,103,104]. Thus far, neutralizing monoclonal antibodies have been found to target four major epitopes on the HIV-1 envelope gp41 and gp120 glycoproteins [105,106,107]. These monoclonal antibodies target the MPER epitope of gp41 (monoclonal antibodies 2F5, 4E10, M66.6, CAP206-CH12 and 10e8) [107,108,109,110,111,112,113,114,115]; the V1V2-glycan of gp120 (PG9, PG16, CH01-04 and PGT 141–145) [116,117,118,119]; the glycan dependent site of the gp120 V3 loop (PGT121–123, PGT125–131 and PGT135–137) [119]; and the CD4-binding site (b12, HJ16, CH103–106, VRC01–03, VRC-PG04, VRC-PG04b, VRC-CH30–34, 3BNC117, 3BNC60, NIH45–46, 12A12, 12A21, 8ANC131, 8ANC134, INC9 and IB2530 [102,114,120,121,122,123,124,125,126,127,128]. Monoclonal antibody 2G12 targets the surface glycans on the outer domain of gp120 that is distinct from the four major epitope target sites described above [102,104,129].

The inovirus display technology has also been applied in vaccination strategies against HIV-1 [130]. However, unlike the successful development of vaccines against non-HIV-1 parasites, these efforts failed. In all published studies (see Table 1), the HIV-1 inovirus-display vaccines were made utilizing the broadly neutralizing antibodies 2F5, 2G12 and b12 [50,52,54,55,59,62]. The first study for the construction of a vaccine against HIV-1 using inovirus display technology was performed in 1993 by Keller *et al.*, using the 447-52D monoclonal antibody to screen a 15-mer RPL [50]. Vaccination of selected recombinant inoviruses in rabbits resulted in the induction of type-specific neutralizing antibodies [50]. A few years later, in 2001, Zwick *et al.* used b12 monoclonal antibody to screen a variety of linear and constrained RPLs [52]. However, the vaccination in mice and rabbits with the selected recombinant inoviruses did not result in the production of b12-like antibodies at detectable levels [52]. The same antibody was used in 2005 by Dorgham *et al.*, in order to screen a 15-mer RPL, and the selected mimotopes were fused to the capsid of inoviruses which were then used to vaccinate mice [54]. The induced antibodies could bind gp160 but they did not have neutralizing potency [54]. In another study in 2007, Wilkinson *et al.* screened a 9-mer and a constrained 10-mer library with antibody 5145A [55]. This was the only case in which the selected mimotopes were fused in to small heat shock protein (HSP) of the archeaon *Methanococcus jannaschii* as a carrier protein. Following vaccination of HSP-mimotopes in rabbits, anti-gp120 antibodies without neutralizing potency were produced [55]. The 2G12 antibody was used for the first time in 2008 by Menendez *et al.* for the screening of a variety of linear and constrained RPLs [59]. Nevertheless, vaccination of selected inoviruses in rabbits induced the production of antibodies that could not bind to gp120 [59]. More recently, in 2011, Rodriguez *et al.* used the 2F5 antibody to screen a 12-mer and a constrained 7-mer RPL [62]. Vaccination in mice and rabbits led to the production of non-neutralizing antibodies [62]. In most of these studies, the use of inoviral vectors resulted in the induction of a specific humoral response. However, the sera of the vaccinated animals did not have broadly neutralizing ability.

Besides monoclonal antibodies, polyclonal sera from HIV-1-infected patients were also used for the screening RPLs (Table 1) [51,53,56,57,58,63]. This approach carries a degree of uncertainty, since it is based on the premise that the polyclonal sera will contain at least one broadly neutralizing monoclonal anti-HIV-1 antibody, meaning that a new neutralizing epitope against it can be isolated from the RPL. It is suggested that long-term non-progressors (HIV-1-infected patients who remain asymptomatic for a long time) are more likely to produce neutralizing antibodies in comparison to AIDS patients, and it is suggested that these neutralizing antibodies in the serum of long-term non-progressors may contribute to the control of viral load [131,132,133]. However, this hypothesis has been questioned [134]. Polyclonal serum for the screening of RPLs was used for the first time in 1999 by Scala *et al.*, who screened both linear and constrained 9-mer RPLs [51]. After that, vaccination of selected inoviruses in mice led to the production of neutralizing antibodies. A few years later, in 2007, Rodriguez *et al.* used polyclonal serum to screen a 7-mer, a 12-mer and a constrained 7-mer RPL [56]. Vaccination in mice induced the production of antibodies that could bind to gp41, but no information was provided about their neutralization potency [56]. Additionally, in 2007, Humbert *et al.* used polyclonal serum to screen a 7-mer, a 12-mer and a constrained 7-mer RPL [57]. Vaccination of selected recombinant inoviruses in mice induced the production of neutralizing antibodies [57]. The screening of the same RPLs using polyclonal serum from a monkey infected with a Simian-Human Immunodeficiency Virus (SHIV) was performed by the same group. In this study, vaccination in mice was performed using the prime-boost strategy: DNA vaccine as prime (encoding gp160) and a cocktail of recombinant inoviruses as boost. The result was the induction of neutralizing antibodies [58]. In 2013, Gazarian *et al.* screened a linear 12-mer and constrained 7-mer RPLs using sera from HIV-infected individuals [63]. Vaccination in rabbits resulted in the production of antibodies that bind gp160 [63]. In 2001, non-human primates were used by Chen *et al.*, as an animal model for vaccination with recombinant inoviruses [53]. This group performed screening of a 9-mer and a constrained 9-mer RPL with polyclonal serum isolated from an infected donor. After that, the vaccination of selected inoviruses was performed in rhesus macaques. As a result, sera from the vaccinated macaques exhibited neutralizing activity. Furthermore, the vaccinated macaques were not protected from infection, but four out of five animals were able to control the viral load after challenge against the pathogenic SHIV89.6PD virus. This was a very important result, which underlines the potential of the method. Additionally, no specific CTL response was detected, implying that the control of viral load was an exclusive result of humoral response [53].

Since the first attempt to induce the production of anti-HIV-1 neutralizing antibodies using inovirus-based vaccines, all efforts to date have not led to the production of broadly neutralizing antibodies in vaccinated animals. This failure could be to a certain extent explained by the usage of the “old generation” monoclonal antibodies (2F5, 2G12 and b12) which were shown to demonstrate autoreactivity properties *in vitro* studies [135,136,137,138,139]. In 2010, Verkoczy *et al.* demonstarted in an *in vivo* study that Pre-B cells expressing 2F5-like antibodies were unable to maturate in mice, suggesting a triggering of immunological tolerance due to the autoreactive properties of the 2F5-like antibody [140]. Collectively, these studies implied that the screening RPLs using 2F5, 2G12 and b12 could result in inoviral-based vaccines that could trigger immunological tolerance in vaccinated animals. It is important to note, however, that the lack of autoreactivity properties for several of the “next generation” broadly neutralizing antibodies (10e8, PG9, PG16, VRC01–03, VRC-PG04, VRC-PG04b, and VRC-CH30–34) could solve the autoreactivity problems encountered by 2F5, 2G12, and b12. 

## 5. IAVs Induce Strong Cellular Immune Responses

Despite the fact that the recent isolation of new broadly neutralizing antibodies against HIV-1 has focused the attention of HIV-1 vaccine development on the induction of a humoral anti-HIV-1 response, recent results underline the importance of a cellular anti-HIV-1 response as well [141,142]. The experimental results concerning inovirus-based vaccines in non-HIV-1 diseases prove that inoviruses can also induce a strong specific cellular response (Table 1 and Table 2); this property makes them great candidates as vectors for HIV-1 vaccine design. The ability of inoviruses to induce a cellular immune response was first demonstrated in 2000 by DeBerardinis *et al.* [143]. In this work, recombinant inoviruses carrying the RT2 epitope and the pep23 epitope of HIV-1 reverse transcriptase in gp8 could induce specific cellular responses in human cell lines *in vitro* and in mice *in vivo* against the RT2 peptide. It is interesting that without the pep23 epitope, the cellular response was undetectable. This indicates that the pep23 is a CTL epitope that is necessary for cellular response, possibly because it enables internalization of the recombinant inovirus into the APCs [143]. Therefore, a question arises of whether an epitope fused to the surface of the inovirus can induce a cellular or a humoral response or both. It is suggested that the type of immune response caused by an epitope fused on the surface of the inovirus is dependent on the length and sequence of the peptide [143]. In 2003, Gaubin *et al.* demonstrated that FITC-labeled fd virions can be internalized in human EBV-B cell lines and the fd virions are successively degraded and targeted both to MHC class I and class II antigen-processing pathways [144]. This was confirmed after endocellular localization of the labeled virions with confocal microscopy. This experiment showed that the inoviruses could be internalized in APCs even without carrying a CTL epitope, but in very low rate. For *in vivo* experiments however, it is possible that the requirement of a CTL epitope is critical for the induction of a cellular response [144]. In 2011, Sartorius *et al.* showed that a hybrid fd virion with the anti-DEC-205 scFv antibody fragment fused on gp3 and the OVA_257–264_ antigenic epitope fused on gp8 can be internalized in human dendritic cells through a specific interaction between the anti-DEC205 scFv and the DEC-205 receptor [145]. In addition, inoculation of mice with the hybrid virions induced a specific cellular response against the OVA_257–264_ epitope [145]. This important characteristic of inoviral vectors to induce a cellular response was reported in only two studies aimed at developing a HIV-1 inoviral vaccine, possibly due to the complexity of detecting a cellular response and also because it is a labor intensive and time-consuming process. In 2001, Chen *et al.* attempted to detect a cellular response, but such a response was not induced in that experiment [53]. A more recent research study related to HIV-1, which clearly demonstrates the ability of inoviruses to induce strong cellular response, was performed in 2009 by Pedroza-Roldan *et al.* [60]. In this effort, an immunodominant CTL epitope of the V3 loop of gp120 (residues 311–320) was expressed as fusion to gp8 of an M13 inovirus. A random peptide library was created by inserting mutations in certain positions of this epitope. A cocktail of inoviruses carrying the V3 loop epitope or variations of this epitope were used for the vaccination of mice and, as a result, a CTL response was induced. The most important result of this study was that the immunization induced long-lasting memory T-cell responses, which were detected seven months after a single immunization [60]. The same vaccination also induced a strong humoral response, since the sera of vaccinated mice could neutralize five out of ten pseudoviruses from a panel [61]. All the above experiments clearly show that the inoviruses are capable of inducing a specific cellular immune response: the ability of the inoviral vectors to induce both arms of adaptive immunity is unique and it could prove to be valuable in the development of a successful HIV-1 vaccine.

In the general field of HIV-1 vaccine design, all studies for the production of anti-HIV-1 broadly neutralizing antibodies through vaccination with either soluble peptides or viral vectors have been unsuccessful. For this reason, some efforts have been directed to the induction of cellular immune response [146,147]. In recent years, in HIV-1 vaccine phase I and phase II clinical trials, adenoviral vectors have been used in order induce a cellular immune response in HIV-1 vaccinated individuals [148,149,150,151]. However, there are concerns about the safety of these viruses. In 2007, the large-scale phase IIB Merck trial was abruptly terminated because there was evidence that the individuals vaccinated with adenovirus rAd5 vector (expressing gag, pol and nef) became more vulnerable to HIV-1 infection in comparison to controls. It was suggested that the group with the increased risk of being infected with HIV-1 consisted of individuals who were Ad5 seropositive [152]. Other eukaryotic viruses that infect other species and do not replicate in human cells were also tested as candidates HIV vaccine carriers and in theory are safer. For example, the canarypox vector was used in the RV144 phase III clinical trial, the only clinical trial that had positive results to date, offering partial protection (31%) to vaccinated individuals in comparison with control [152,153]. Apart from safety reasons, the use of adenovirus-based vectors has not been protective. The recent HVTV 505 phase IIB trial that used adenovirus rAd5 as a boost and DNA as prime for vaccination of 2504 human volunteers was abandoned as futile [150]. Recently, a rhesus macaque cytomegalovirus (RhCMV) vector successfully induced a persisting CTL response in rhesus macaques that strongly protected the vaccinated animals from challenge against the pathogenic SIVmac239 strain. Importantly, this study, 50% of the vaccinated animals reduced the viral load to undetectable levels [141,142]. However, the design of a human version of this CMV vector could impose safety risks, since the human CMV is a persistent and pathogenic human virus [146]. Therefore, various types of eukaryotic viral vectors are currently under investigation. These are reviewed elsewhere [154,155]. However, the use of a eukaryotic virus is accompanied by serious safety concerns. As an alternative, the use of prokaryotic viruses such as inoviruses, may be utilized which have a decisively lower safety risk to humans. Even if inoviruses could infect a eukaryotic cell, the assembly of the new prokaryotic virions cannot take place without the specific conditions that exist in the inter-membrane area of the *E.* c*oli* and without the presence of the specific *E.* c*oli* enzyme leader peptidase that does not exist in human cells [156,157]. Furthermore, there was a phase I case study in 2006 where fd inoviruses were intravenously infused in humans (for purposes unrelated to vaccination), causing no side effects or even allergic reactions in any of the eight volunteers. To our knowledge, this is the only case where inoviruses were infused in humans [158]. Therefore, in contrast to other viral vectors, the use of inoviruses does not impose a major safety risk to humans. This characteristic of inoviral vectors along with their capability to trigger both cellular and humoral immune responses makes them an attractive option as vaccine vectors.

## 6. Conclusions

During the last two decades, inoviral vectors have been used in the development of vaccines against various infectious parasites and against non-infectious diseases like cancer and Alzheimer’s with promising results. While the applications of inovirus display technology in vaccine design against non-HIV-1 diseases have been mostly successful, the design of a HIV-1 vaccine development has so far been disappointing. A major obstacle has been the use of neutralizing monoclonal antibodies plagued with autoreactivity properties. Screening of RPLs with these antibodies resulted in the isolation of peptides that, as vaccine antigens, were unsuccessful in inducing a specific humoral response that would produce neutralizing antibodies. The recent isolation of antibodies such as VRC01 and 10E8 with more neutralizing breadth and potency without autoreactivity properties than the previously utilized antibodies may overcome this obstacle [115,124]. Particularly, the induction of VRC01-like and 10E8-like antibodies could be a feasible target, since these antibodies also seem to be produced from a significant percentage of the HIV-1-infected population [115,126,127]. While humoral responses have been well documented, cellular responses have not been assessed in most studies for the design of a HIV-1 vaccine using inoviruses. The only study in which a cellular anti-HIV-1 response was detected also reported a successful induction of a long-lasting memory CTL response seven months after a single vaccination in mice with inoviral particles [60], demonstrating that the induction of cellular immunity against HIV-1 using inoviruses is feasible. IAVs are advantageous in that they can induce both arms of adaptive immunity. This finding could therefore be of importance in future efforts for the design of a HIV-1 vaccine.

Moreover, IAVs have unique characteristics compared to other viral vectors: they are stable, they can display a peptide in multiple (from few to thousands) copies on their surface and such constructs are very immunogenic without the use of an adjuvant. In addition, IAVs allow the immune system to focus on a specific epitope of interest instead of the whole protein, which is of great importance, since an important aspect for successful HIV-1 vaccine design is to focus on the induction of neutralizing antibodies against the specific neutralizing epitopes while at the same time avoiding the induction of ineffective antibodies against the numerous non-neutralizing epitopes of the HIV-1 glycoproteins, which act as decoys for the immune system. Ideally, an effective HIV-1 vaccine should be able to stimulate both humoral and cellular immune responses. Recently, adenoviral vectors were tested in clinical trials as HIV-1 vaccine carriers in order to induce cellular immunity, but they were shown to impose serious health risks for humans. On the other hand, IAVs are able to induce cellular immunity and at the same time they have been demonstrated to be safe for administration in animals and humans. These characteristics of IAVs, conferred by their structural and biological properties, make them effective antigen display vectors that can induce strong and specific humoral and cellular immune responses against the displayed antigen. These properties of IAVs along with newly discovered broadly neutralizing anti-HIV-1 antibodies, pave the way for the development of an effective HIV-1 vaccine.
